# DNA methylation downregulated ZDHHC1 suppresses tumor growth by altering cellular metabolism and inducing oxidative/ER stress-mediated apoptosis and pyroptosis

**DOI:** 10.7150/thno.45631

**Published:** 2020-07-25

**Authors:** Xin Le, Junhao Mu, Weiyan Peng, Jun Tang, Qin Xiang, Shaorong Tian, Yixiao Feng, Sanxiu He, Zhu Qiu, Guosheng Ren, Ailong Huang, Yong Lin, Qian Tao, Tingxiu Xiang

**Affiliations:** 1 Key Laboratory of Molecular Oncology and Epigenetics, The First Affiliated Hospital of Chongqing Medical University, Chongqing, China.; 2 MOE Key Laboratory of Molecular Biology for Infectious Diseases, Department of Infectious Disease, Chongqing Medical University, China.; 3 Lovelace Respiratory Research Institute, Albuquerque, New Mexico, USA.; 4 Cancer Epigenetics Laboratory, Department of Clinical Oncology, State Key Laboratory of Translational Oncology, Sir YK Pao Center for Cancer and Li Ka Shing Institute of Health Sciences, The Chinese University of Hong Kong, Hong Kong.

**Keywords:** ZDHHC1, ER stress, Oxidative stress, CYGB, zinc finger protein

## Abstract

Cancer progression is an intricate biological process profiled by not only unscheduled proliferation, but also altered metabolism mechanisms. In this article, we introduced a novel tumor suppressor gene (TSG), Zinc Finger DHHC-Type Containing 1 (ZDHHC1, also known as ZNF377), frequently silenced due to epigenetic modification among various cancers, which exerts significant anti-tumor effects through metabolic regulation.

**Methods:** Quantitative reversed-transcription PCR (qRT-PCR), reverse transcription PCR (RT-PCR) and Western blot were employed to demonstrate transcriptional and protein levels of targeted regulators. Methylation of ZDHHC1 promoter was detected by bisulfite genomic sequencing (BGS) and methylation specific PCR (MSP). Proteomics were analyzed by isobaric tags for relative and absolute quantitation (iTRAQ) and gas chromatography-mass spectrometry (GC-MS) were utilized for metabolomics analysis. Cellular functions were examined via corresponding approaches. Nude mice were used for xenograft tumor models. Indirect immunofluorescence staining was utilized to obtain precise location and expression of target proteins. Oxidative and ER stress indicators were detected using specific kits.

**Results:** We found that ZDHHC1 expression was frequently silenced in multiple tumor cells and specimens due to methylation. Restoration of ZDHHC1 expression can curb cancer cell progression via stimulating apoptosis and cell cycle arrest, repressing metastasis, and reversing EMT transition and cell stemness. ZDHHC1's salient anti-tumor abilities were recognized *in vivo* as well. Metabolomic and proteomic analyses predicted inhibitory role of ZDHHC1 in glucose metabolism pathways in a CYGB-dependent manner, and in pentose phosphate pathway (PPP), which was validated by examining altered key factors. Moreover, we unraveled that ZDHHC1 dedicates to the increment of oxidative stress and endoplasmic reticulum (ER) stress to promote pyroptosis for anticancer purposes.

**Conclusion:** Our study for the first time indicates ZDHHC1 is a potential tumor-suppressor frequently silenced due to promoter methylation, capable of negatively regulating metabolisms of tumor cells while stimulating oxidative stress and ER stress to expedite cell death through induction of pyroptosis and apoptosis, which can be exploited for development of new cancer prevention and therapies.

## Introduction

As the leading cause for premature death, cancer is threatening the health of every one of us, leaving heavy health burden worldwide due to lack of highly-effective prevention and remedy measures 1. Though cancer has historically been considered a disorder of cell biology characterized by cell ceaselessly proliferation and invasion/metastasis, recently presented evidences have suggested that it should also be viewed as a metabolic disease 2. Malignant cells usually undergo altered metabolism such as supporting increased energy requirement and maintaining redox homeostasis in favoring their survival and proliferation 3. Increase of glucose uptake and metabolism such as glycolysis, glucose oxidation or the pentose phosphate pathway (PPP); is advantageous for high energy demand and unlimited growth and proliferation in tumor cells 2, 4, 5.

Carcinogenesis is also a process in which cells resist multiple stresses, including oxidative stress and ER stress during their growth and expansion. Oxidative stress, a state of lost balance between the oxidative and anti-oxidative systems of the cells and tissues, results in the over production of oxidative free radicals and reactive oxygen species (ROS) 2, 6. While heavy oxidative stress associated with high proliferation rates causes severe damage in the cellular components, tumor cells develop mechanisms to scavenge reactive oxygen species (ROS) for maintaining redox homeostasis. It has been revealed that a positive feedback exists between oxidative stress and ER stress, which tightly involves in a variety of physiological and pathophysiological process, interfering with cell homeostasis and apoptosis 7, 8. ER stress has controversial effects on neoplasia, but solid evidences suggest its tumor-inhibitory identity as well 9, 10. Therapeutic strategies targeting metabolic mechanisms in malignant cells have been proven efficacious and are widely studied nowadays 11, 12. Accumulated evidences suggest that excessive ROS generated in oxidative stress process may activate caspase-1 and result in pyroptosis 13. Pyroptosis is a newly discovered member and a consequent programmed death of cancer extermination 14, 15. It is accompanied by inflammatory or immunological stimulations. Distinct from apoptosis, pyroptosis needs the participation of inflammasome, and gasdermin family as executioners. Morphologically, pyroptotic cells exhibit swelling and membrane rupture. It is believed that pyroptosis might play dual roles in terms of tumorigenesis 16, inspiring us to hunt for new treatment targeting pyroptosis alteration. Nowadays, newly developed strategies have been focusing on involving pyroptosis to create a more efficacious way for inhibiting tumor formation and metastasis 17-19. Studies aiming at the switch of apoptosis-to-pyroptosis is gaining attention in cancer therapy.

To gain a full perspective of tumor metabolic change mechanisms, we need to discover the important regulators consistently activated or repressed in tumorigenesis process. Oncogenes and tumor suppressors have been discovered to have crucial roles in cancer-associated metabolism changes as well. Zinc Finger DHHC-Type Containing 1 (ZDHHC1, also known as ZNF377), is located at chromosome 16q22.1 (https://www.ncbi.nlm.nih.gov/gene/29800), and the protein is localized on the ER and membranous structure in the cell 20. As an understudied gene, there are limited reports about biological and cellular roles of ZDHHC1. It has been implicated to promote immune response upon virus infection 21. Methylation of ZDHHC1 promoter has been linked to inflammatory bowel disease (IBD)-associated neoplasia 22.

In this study, we examined the expression of ZDHHC1 in multiple cancers and the cellular functions of ZDHHC1 focusing on the effects on metabolism. We found ZDHHC1 is epigenetically silenced in a portion of cancer, and ectopic ZDHHC1 expression is able to diminish canonical cancer cell aggressiveness, most likely by reducing metabolic activity through promoting oxidative stress, ER stress mediated pyroptosis and apoptosis. Therefore, targeting of ZDHHC1-mediated metabolic signaling pathways may be a promising strategy for cancer prevention and therapy.

## Results

### ZDHHC1 is frequently silenced in tumor due to methylation

**Figure 1:**
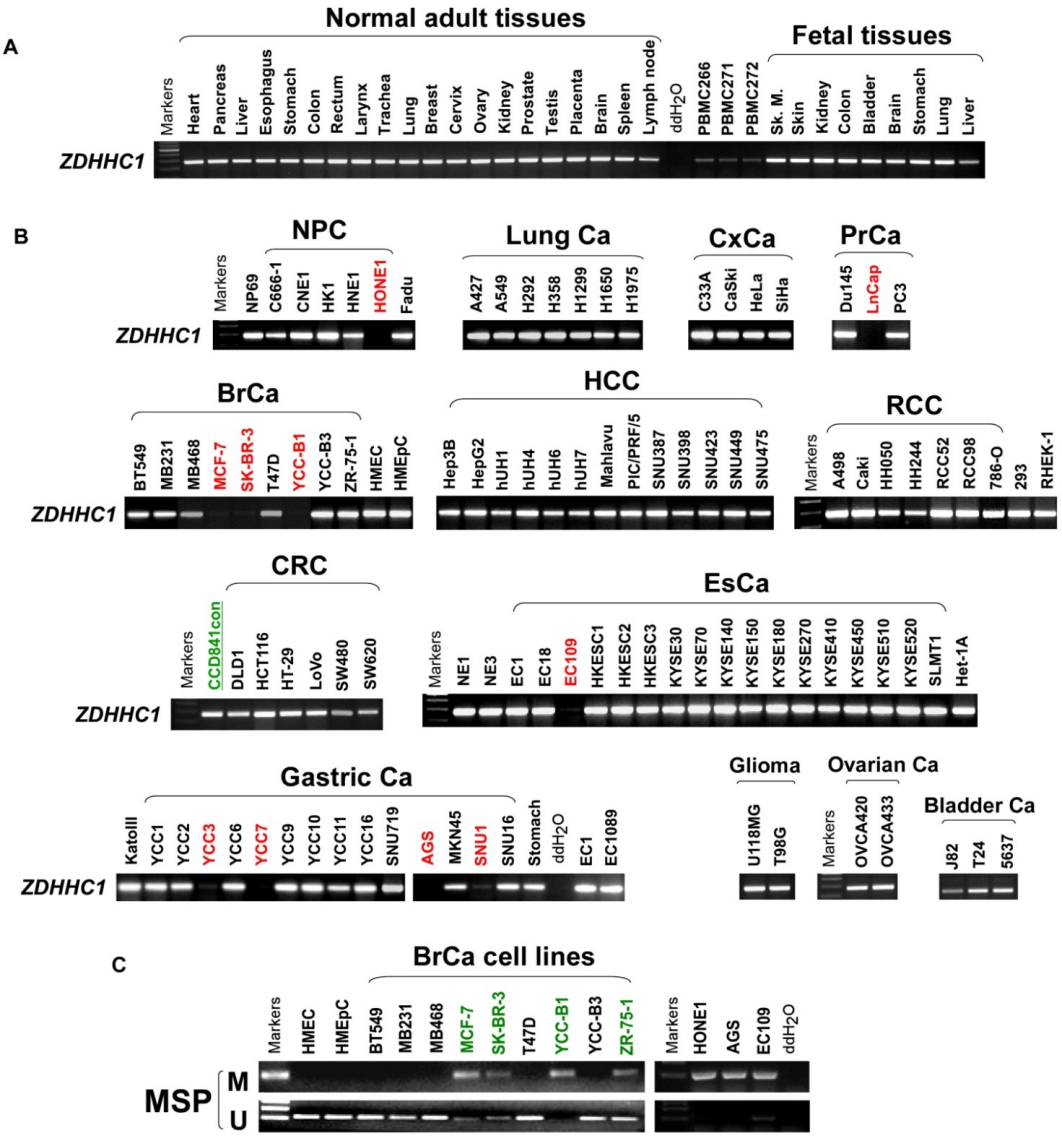
** The expression of *ZDHHC1* in multiple normal tissues and tumor cells.** (**A**). RT-PCR assays demonstrated a wide range expression of *ZDHHC1* among nearly all normal adult tissues and fetal tissues. (**B**). RT-PCR assays showed downregulated or silenced *ZDHHC1* in a number of cancer cell lines. (**C**). The methylation state of the *ZDHHC1* promoter was detected by methylation-specific PCR (MSP).

**Figure 2:**
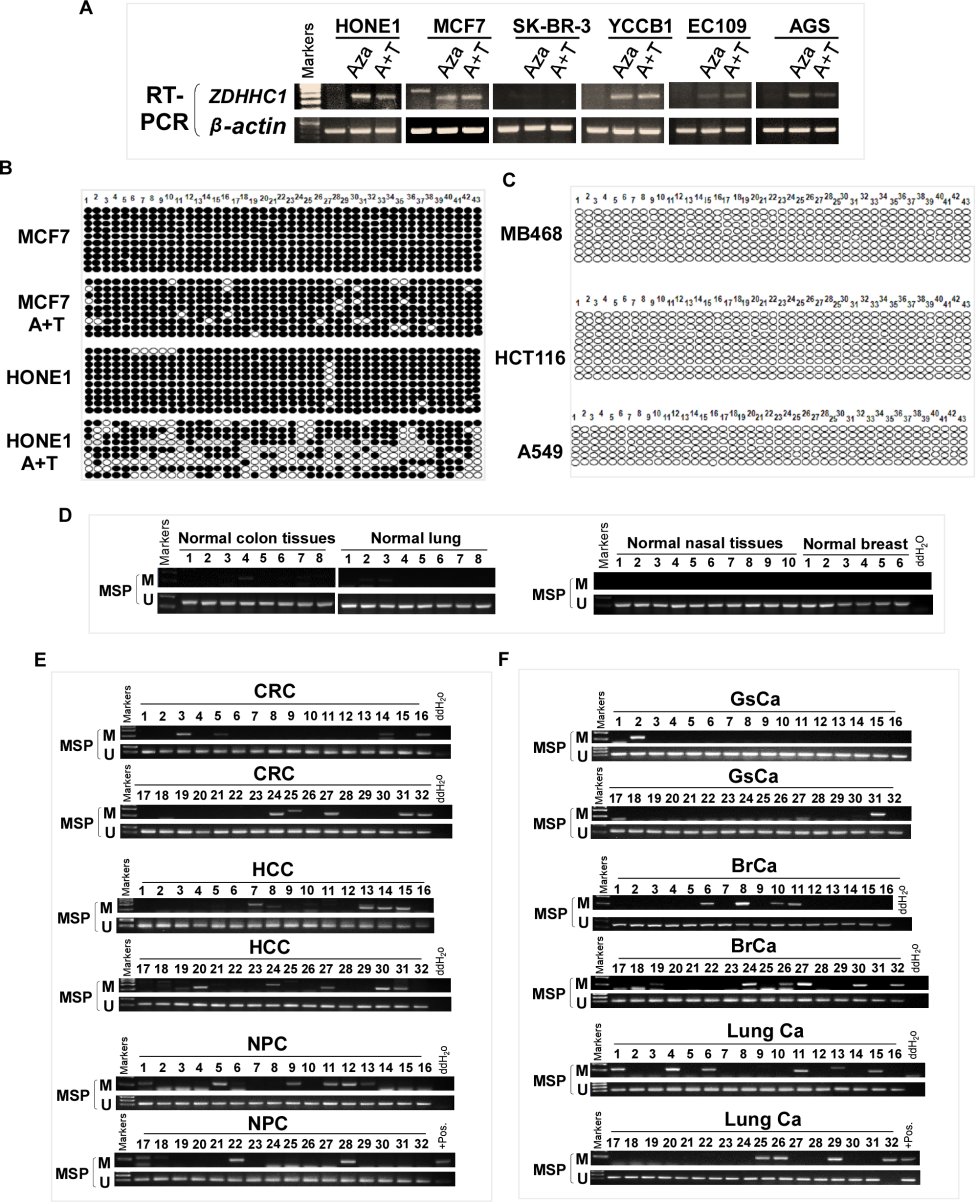
** Promoter CpG Methylation Mediates *ZDHHC1* Downregulation in multiple Cancers.** (**A**). Pharmacologic and genetic demethylation reactivated *ZDHHC1* expression in carcinoma cell lines. Aza (**A**), Trichostatin A (T). (**B-C**). Methylation alleles of *ZDHHC1* demonstrated by BGS in multiple tumor cells, every row of circles indicated an individual promoter allele clone sequenced. Filled circles represented methylated CpG sites, blank ones referred to unmethylated sites. Aza (A), Trichostatin A (T). (**D-F**). The methylation status of *ZDHHC1* in multiple normal and cancer tissues measured by MSP.

ZDHHC1 is ubiquitously expressed in normal adult and fetal tissues (Figure 1A), but is silenced or downregulated in several cancer cell lines (Figure 1B). As tumor suppressor genes (TSGs) tend to be methylated for gene silencing and it was shown that ZDHHC1 promoter methylation was detected in IBD-associated cancer 22, we applied methylation-specific PCR (MSP) and found ZDHHC1 promoter was methylated (Figure 1C). In ZDHHC1-null cells, re-expression of ZDHHC1 was detected after de-methylation treatment (Aza, A+T) (Figure 2A). Bisulfite genomic sequencing (BGS) assay revealed that those ZDHHC1- negative cell lines were proven to carry numerous methylated CpG sites within the promoter region, which can be de-methylated once cells were treated with demethylating agents (Figure 2B). In contrast, the MB468, HCT116 and A549 cells exhibiting ZDHHC1 expression were shown to have no methylation (Figure 2C). Consistent with cell lines, tumor and adjacent normal tissues collected from representative carcinomas (colon, hepatocellular, nasopharyngeal, gastric, breast and lung tumors) were examined for ZDHHC1 promoter methylation with MSP and BGS. A dramatic tendency of methylation on promoters of tumor specimens was detected, comparing with adjacent normal tissues (Figure 2D-E). All these results suggested that ZDHHC1 is frequently silenced in tumor through promoter methylation.

### ZDHHC1 exhibits tumor-suppressive functions in multiple cancer cell lines

**Figure 3:**
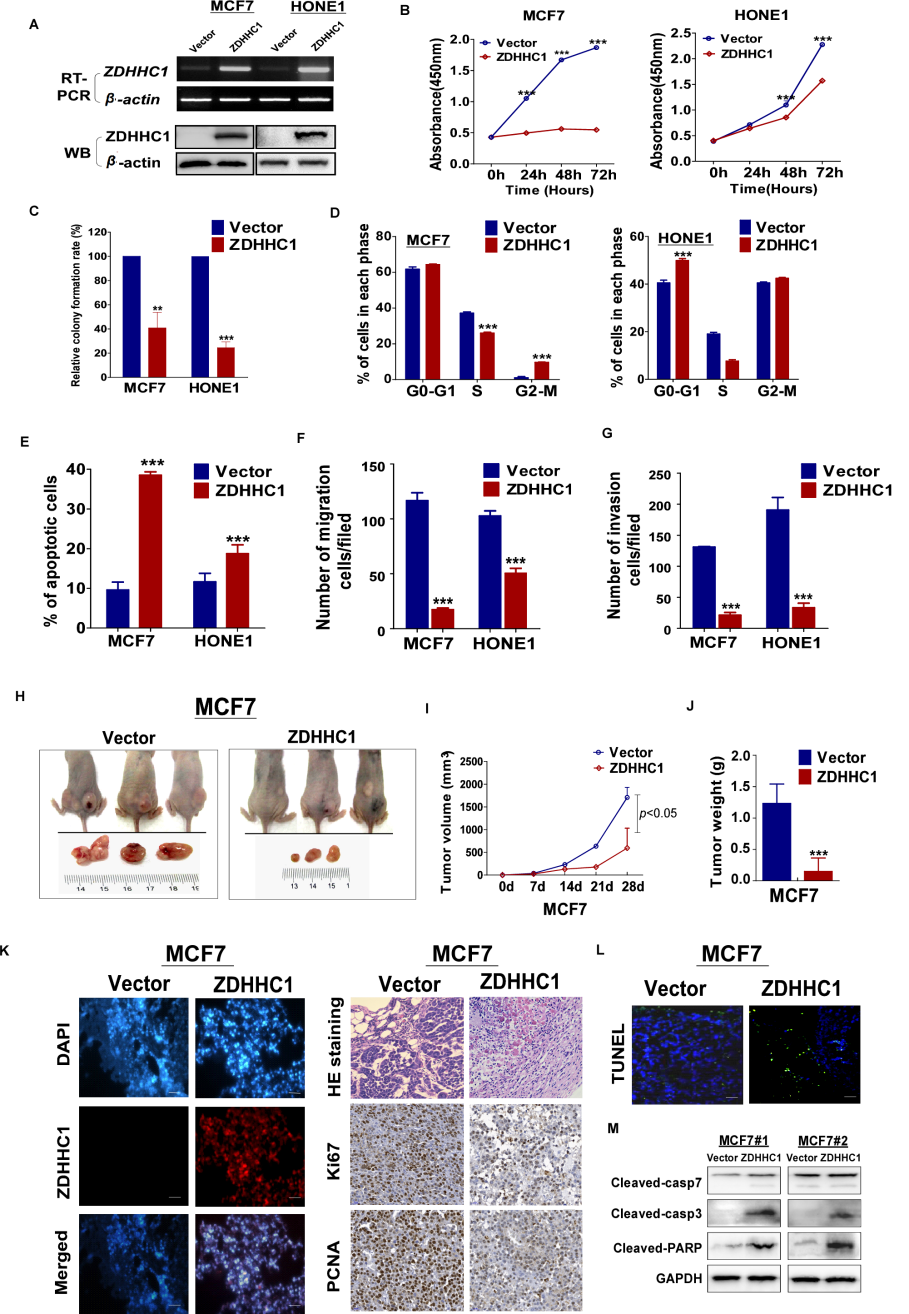
*** ZDHHC1* inhibited growth, migration and invasion of MCF7, and HONE1 cells. Analysis of nude mice xenografts generated by *ZDHHC1*- overexpressing cell.** (**A**). Ectopic expression of ZDHHC1 was confirmed by RT-PCR and Western blot. (**B**). Measurement of cell proliferation for vector- and *ZDHHC1*-transfected carcinoma cells through CCK-8 assay. (**C**). Histogram statistics of CFA. MCF7 and HONE1 cells transfected with* ZDHHC1* or empty vector were used for colony formation assay to measure proliferation rates. (**D**). Histogram for flow cytometry analysis of cell cycle progression. (**E**). Histogram for flow cytometry analysis of cellular apoptosis. Vector and *ZDHHC1* transfected MCF7 and HONE1 cells were double stained with Annexin-V-FITC and PI for apoptosis measurement. (**F**). Histogram of quantified migration cells/field, Transwell assays measuring cell migration ability, using two cells transfected with Vector or *ZDHHC1* 24 h after seeding. (**G**). Histogram of quantified invasion cells/field, Transwell assays measuring cell invasion ability, using MCF7 and HONE1 cells transfected with Vector or* ZDHHC1.* Three independent experiments were carried out. **: *p* < 0.01; ***: *p* < 0.001. (**H-J**). Tumor growth was significantly stalled in nude mice xenograft model injected with MCF7 cells (n=6 /group). (H) Representative images of xenografts before and after resection in Vector and *ZDHHC1* groups. (I) Line graphs of tumor volume at various time points after injection. (J) Histogram of tumor weight by the time of resection. (**K**). (left) Representative immunofluorescent staining of *ZDHHC1* in MCF7 xenografts. Red for *ZDHHC1*, blue for nucleus DNA counterstained with DAPI. (right) H&E staining, and immunohistochemistry staining of Ki67 and PCNA in MCF7 xenografts overexpressing *ZDHHC1* or vector. (**L**) TUNEL assays of MCF7 xenografts overexpressing *ZDHHC1* or vector. Green for fluorescein-dUTP, and blue for DNA counterstained with DAPI. *p < 0.05, ** *p* < 0.01, ***p < 0.001. (**M**) Western blot results of critical apoptosis indicators in the xenograft tissues (Cleaved caspase-7, caspase-3 and PARP). GAPDH was taken as internal control.

To investigate the role of ZDHHC1 in human cancer cells, we ectopically expressed ZDHHC1 in ZDHHC1-negative HONE1 and MCF7 cell lines by stable transfection. The ectopic expression of ZDHHC1 was confirmed by RT-PCR and Western blot (Figure 3A). By CCK-8 and colony formation assays, we found ZDHHC1 effectively inhibited cell proliferation (Figure 3B-C, Figure S1A). Cell cycle analysis showed that ZDHHC1 expression resulted in cell arrest at the G0/G1 phase in HONE1 cells. In MCF7 cells, cells were accumulated in the G2/M phase, possibly due to cell type-specific differences (Figure 3D, Figure S1B). ZDHHC1 expression induced apoptosis in MCF7 and HONE1 cells, which was shown by Annexin V-FITC staining and transmission electron microscopy (Figure 3E, Figure S1C-D). Furthermore, ZDHHC1 inhibited cellular migration and invasion, which was detected by Transwell and wound healing assays (Figure 3F-G, Figure S1E-G). Consistently, knockdown of ZDHHC1 in the ZDHHC1-positive A549 cells stimulated cell proliferation and cell cycle progression, decreased apoptosis, and promoted cell migration and invasion (Figure S2A-E). Altogether, ZDHHC1 exerted the capacity to (i) suppress proliferation, (ii) induce apoptosis, and (iii) suppress migration and invasion, which is generally a critical feature of tumor suppressor gene in tumor cells.

### ZDHHC1 suppresses tumor growth *in vivo* in a xenograft tumor model

We next validated the tumor suppressing role of ZDHHC1 *in vivo* with a nude mice tumor xenograft model. The weight and volume of tumors were significantly lower in tumors derived from ZDHHC1-transfected cells compared to those derived from cells transfected with the control vector (Figure 3H-J). Immunohistochemistry (IHC) staining of the xenograft sections showed that expression of the proliferation markers Ki67 and PCNA was notably decreased while TUNEL staining of the xenograft sections showed an increased level of apoptosis in the ZDHHC1 group (Figure 3K-L). Considering apoptosis is best described by activated caspases represented by caspase 3 and 7, we performed Western blot to testify our conclusion furthermore. It was plain to see that cleaved caspase-3, 7 and PARP all upregulated in ZDHHC1 originated xenograft tumor in comparison of control group (Figure 3M). Collectively, these results confirmed that ZDHHC1 is able to suppress tumor growth *in vivo*.

### Ectopic ZDHHC1 expression reverses epithelial-mesenchymal transition (EMT) and cell stemness

**Figure 4:**
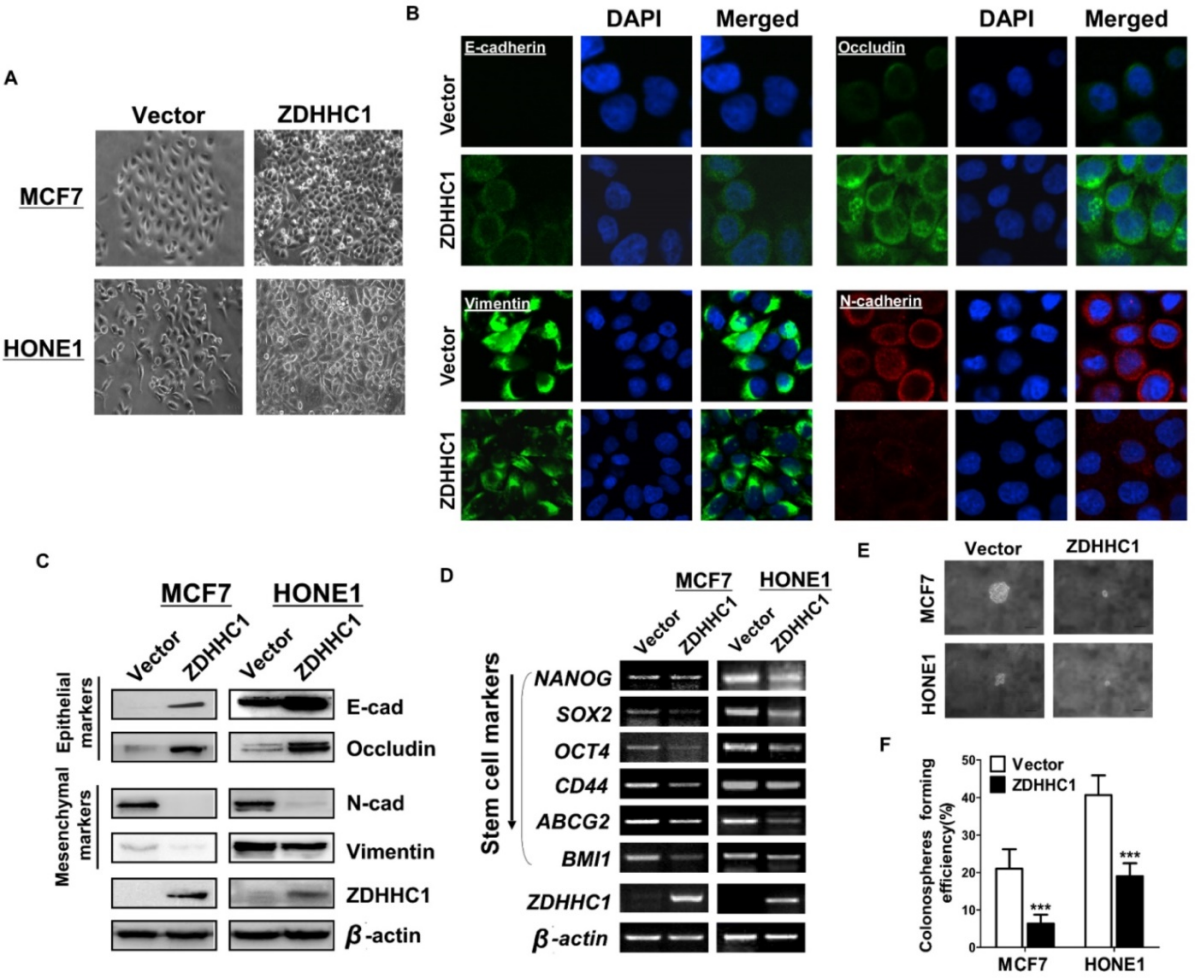
** Ectopic *ZDHHC1* inhibited EMT and disrupted stemness biomarkers in carcinoma cells.** (**A**). Morphology changes of MCF7 and HONE1 cells observed under phase contrast microscopy. (**B**). Immunofluorescent staining of epithelial markers (E-cadherin, Occludin) and mesenchymal markers (N-cadherin, Vimentin) in MCF7 cells transfected with* ZDHHC1* or Vector. (**C**). Western blot assays of epithelial markers (E-cadherin, Occludin) and mesenchymal markers (N-cadherin, Vimentin) in MCF7 and HONE1 cells transfected with *ZDHHC1* or Vector. β-actin was taken as internal control. (**D**). RT-PCR assays of cancer stemness markers (NANOG, SOX2, OCT4, CD44, ABCG2 and BMI1) in MCF7 and HONE1 cells, with *ZDHHC1* or empty vector transfected. β-actin was taken as control. (**E-F**). Spheroid formation assay showed that *ZDHHC1* decreased the size of spheres formed by MCF7 and HONE1 cells. ***p < 0.001.

We then investigated how ZDHHC1 suppresses cancer cell migration and invasion. Morphologically, ectopic ZDHHC1 expressing cells were prone to intimately stick together, rather than to spread around, resembling mesenchymal-epithelial transition (MET) (Figure 4A). With immunofluorescent staining and Western blot assays, we confirmed that ZDHHC1 upregulated the expression of the epithelial markers E-cadherin and Occludin, and downregulated the expression of the mesenchymal markers Vimentin and N-cadherin (Figure 4B-C). Considering the intimate connection between EMT and cancer stemness, we further examined the expression of six key biomarkers of cancer stemness (NANOG, SOX2, OCT4, CD44, ABCG2, BMI1). All these factors showed decreased expression in ZDHHC1 overexpressing cells (Figure 4D), indicating ZDHHC1 was capable of suppressing stemness of tumor cells. ZDHHC1 also lowered the spheroid forming rate of cancer cells in spheroid forming assay (Figure 4E-F), supporting that ZDHHC1 functions as a tumor suppressor that restrains EMT and cell stemness in human cancers.

### ZDHHC1 regulates glucose metabolism in a CYGB-dependent way

To get more insights of the possible anti-tumor mechanisms of ZDHHC1, we used 8-plex isobaric tags for relative and absolute quantitation (iTRAQ) to identify altered pathways in MCF7 cells bearing stable ZDHHC1 transfection. The results showed expression of 33 proteins, including cytoglobin (CYGB) and glucose-6-phosphate dehydrogenase (G6PD), was significantly changed after ectopic ZDHHC1 expression (Figure S3A, Figure S4). Particularly, the positive correlation between ZDHHC1 and CYGB was confirmed by bioinformatics (Figure S5A). qRT-PCR also revealed that CYGB expression was increased in ZDHHC1 expressing cells (Figure S5B). Proteomic analysis revealed that metabolic pathway-associated proteins exhibited the greatest alterations in ectopic ZDHHC1 expressing cells. The main altered pathways were related to glucose metabolism (metabolic, pyruvate, propanoate, and carbon metabolism, gluconeogenesis and glycolysis pathways), suggesting that ZDHHC1 regulates glucose metabolism pathways (Figure S3B). In a previous study, we revealed that CYGB inhibits glucose transporter 1 (GLUT1)-mediated glucose intake and metabolism pathways mediated by hexokinase 2 (HXK2) and lactate dehydrogenase B (LDHB) 23.

Furthermore, iTRAQ revealed that similar glucose metabolism pathways were altered in ectopic CYGB expressing cells (Figure S3C). Therefore, we investigated if the tumor-suppressive effects of ZDHHC1 work through the CYGB-dependent glucose metabolism regulation pathway. Indeed, metabolic alterations were detected in ZDHHC1 or CYGB overexpressing cells by GC-MS and 18 proteins were regulated by both of them (Figure S3D). Principal component analysis (PCA), partial least squares discriminant analysis (PLS-DA), and orthogonal partial least squares discriminant analysis (OPLS-DA) evaluations showed ectopic ZDHHC1 or CYGB expression significantly changed levels of glucose metabolites, supporting that the glucose metabolism pathways in ZDHHC1-transfected cells were altered (Figure S5C) 23. Decreased levels of intracellular glucose and its metabolites, including glucose-6-phosphate and pyruvic acid, were observed (Figure S5D). These results suggested that ZDHHC1 suppresses cancer through inhibiting glucose metabolism-related pathways. Furthermore, the protein levels of GLUT1, HXK2, and G6PD, which play an important role in glucose metabolism, were measured. The results showed a decrease in the levels of all three factors, confirming ZDHHC1 inhibits glucose metabolism (Figure S5E).

**Figure 5:**
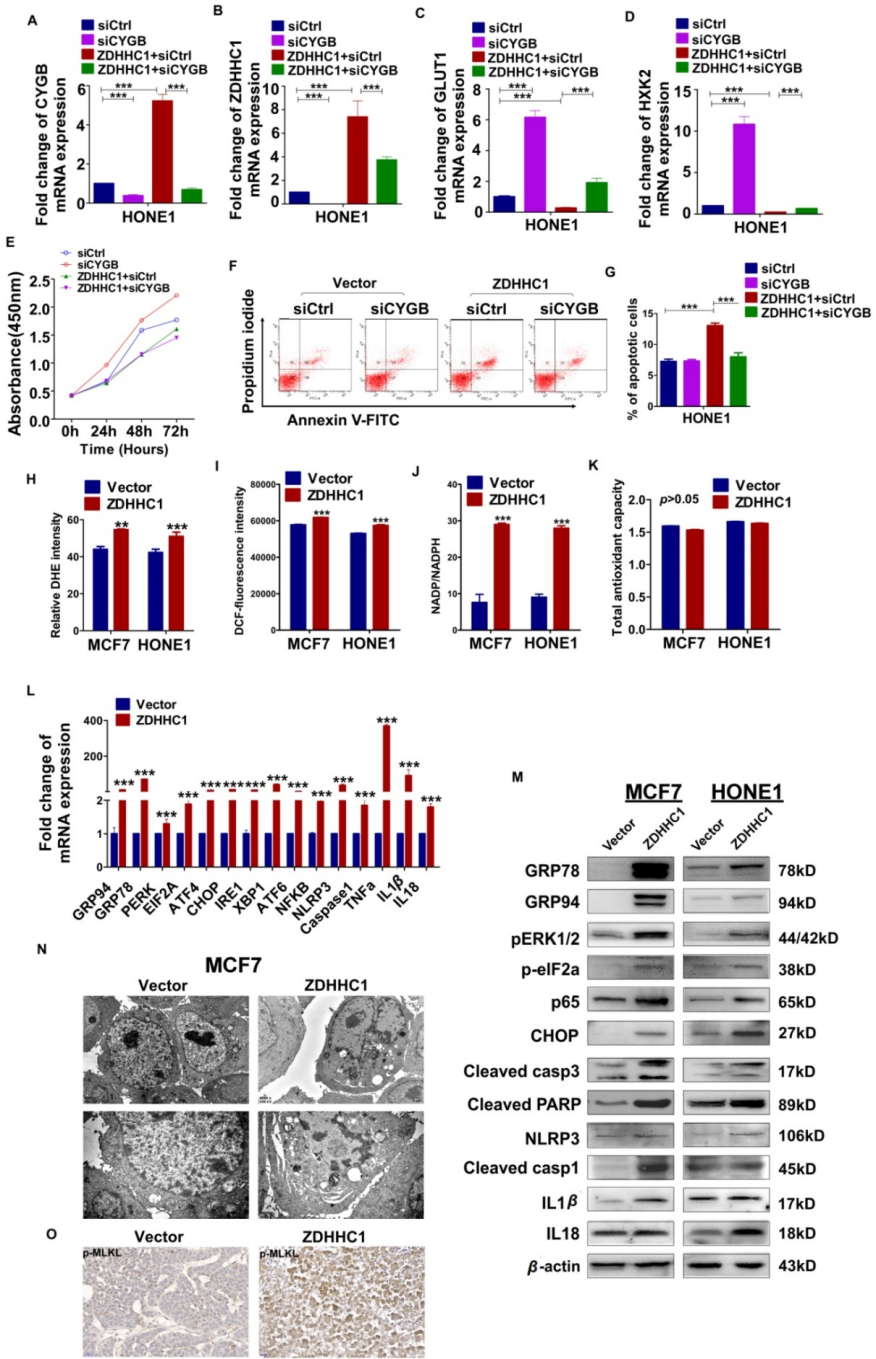
** Effects of ectopic ZDHHC1 expression on proliferation, apoptosis and glucose metabolism in a CYGB-dependent way. Effects of ectopic* ZDHHC1* on ER stress and oxidative stress and thereafter stimulated pyroptosis and apoptosis.** (**A**). The efficiency of CYGB knockdown was confirmed by qPCR. (**B-G**). Effects of *ZDHHC1* overexpression and knock-down of *CYGB* on proliferation, apoptosis and glucose metabolism in carcinoma cell lines. ** *p* < 0.01, ***p < 0.001. (**H**). Intracellular superoxide levels were detected with dihydroethidium (DHE). (**I**). Intracellular ROS was detected by means of oxidation-sensitive fluorescent probe (DCF). (**J**). NADPH oxidase activity was assessed. (**K**). Total antioxidant capacity in the cell was assessed by (T-AOC Assay Kit). (**L-M**). ER stress pyroptosis and apoptosis related markers were detected by quantitative PCR and Western blot. The above experiments were all conducted with MCF7 and HONE1 cells transfected with ZDHHC1 or Vector, respectively. (**N**). Morphology of MCF7 cell undergoing pyroptosis was observed under electronic microscopy. (**O**). Immunohistochemistry staining of pMLKL in MCF7 xenografts overexpressing *ZDHHC1* or vector.

Moreover, we focused succeeding works on whether transfected cells with siRNA-CYGB lose ZDHHC1's anti-tumor effects. qRT-PCR was applied to measure the efficiency of CYGB knockdown (Figure 5A). Indeed, in vector-HONE1 cells, transfection of siRNA-CYGB led to downregulation of ZDHHC1 and upregulation of GLUT1 and HXK2 mRNA compared to siRNA control. In contrast, ZDHHC1 expression decreased levels of GLUT1 and HXK2. Simultaneously, in ZDHHC1-HONE1 cells, knockdown of CYGB partially reversed expression of GLUT1 and HXK2 (Figure 5B-D). The rescue experiment further verified that the knock-down of CYGB could partially reverse the ability of ZDHHC1 overexpression to inhibit the proliferation and induce apoptosis in cancer cell lines (Figure 5E-G). The results furtherly suggested that ZDHHC1 inhibits glucose metabolism in cancer cells at least in part through the CYGB-mediated glucose metabolism pathway.

### ZDHHC1 promotes pyroptosis and apoptosis by oxidative stress and ER stress-mediated inducement

Previous studies have reported that CYGB protects cells from oxidative stress 24. To investigate whether ZDHHC1 also impacts oxidative stress in tumor cells, a series of specific kits were employed to determine the Reactive Oxygen Species (ROS), the superoxide (O_2_^-^) content, the NADP^+^/NADPH ratio, and total antioxidant capacity (TAC). The results showed that the ROS and O_2_^-^ content and the NADP^+^/NADPH ratio increased in ZDHHC1-expressing cells, while the TAC level did not significantly change (Figure 5H-K). ER stress can result from many types of stimulations including oxidative stress that cause misfolded protein accumulation in the ER, resulting in either cell survival or death depending on the extent of cellular damage 9, 10. In the present study, we found that ZDHHC1 upregulated several target genes that are considered key factors of ER stress and the unfolded protein response (UPR) pathway (Figure 5L-M).

Since it has been shown that oxidative stress and ER stress can promote pyroptosis 25-27, we further investigated whether this pathway was activated in ZDHHC1 overexpressing cells. The qRT-PCR and Western blot analysis revealed that the mRNA and protein levels of the nucleotide-binding oligomerization domain-like receptor family, pyrin domain-containing 3 (NLRP3), caspase-1, IL-1β and IL-18 were increased in ZDHHC1 overexpressing cells compared with control cells (Figure 5L-M). Furthermore, transmission electron microscopy revealed that some of the ZDHHC1-expressing cells exhibited pyroptotic morphology: reduced membrane integrity, cell swelling, and lysis (Figure 5N). It has been established that necroptosis is also a programmed necrosis with similar features to pyroptosis 28. Moreover, it is highlighted necroptosis functions in apoptosis-resistant tumor cells as “fail-safe” insurance 29, providing a great reference value for us to test if conversion arisen by ZDHHC1 also includes necroptosis. Since MLKL (mixed lineage kinase domain-like pseudokinase) is the executioner and the most exclusive biomarker for necroptosis 30, we tested its active form, phosphorylated MLKL (p-MLKL) in xenograft nude mice tumor tissues via immunostaining. Results showed a clear disparity where tumor derived from ZDHHC1 overexpressing cells manifested pronouncedly higher levels (Figure 5O). Together, ZDHHC1 is proved to also induce necroptosis, carving a propitious tumor cell demise mode.

**Figure 6:**
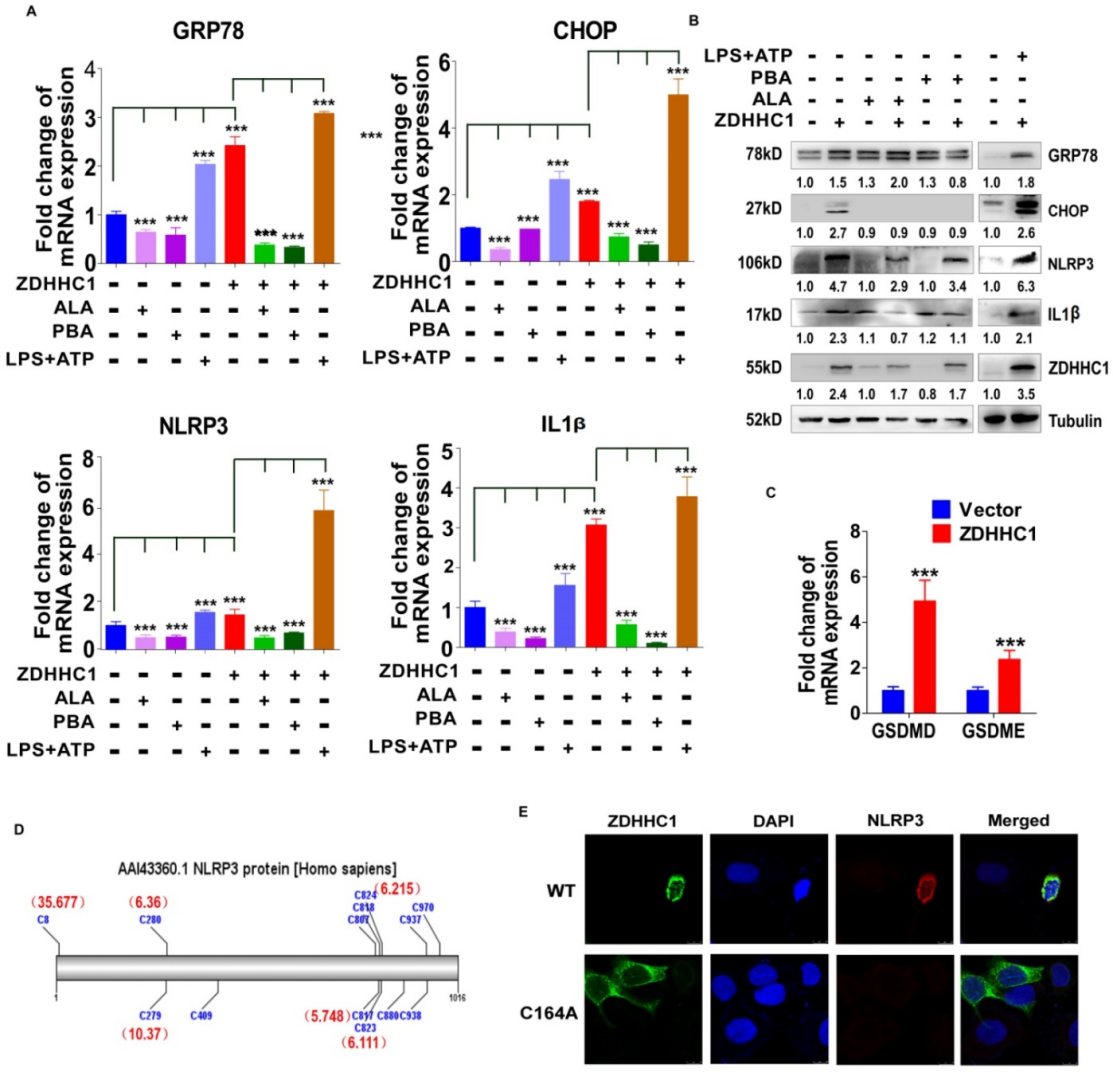
***ZDHHC1* promoted NLRP3 expression is dependent of its enzymatic activity.** (**A-B**) ER stress and pyroptosis markers alteration after drug treatment for separate intentions. GRP78, CHOP, NLRP3 and IL-1β levels were captured by qPCR and Western blot. (**C**). GSDMD and GSDME were detected by quantitative PCR. (**D**). Multiple cysteine residues within NLRP3 are likely to be palmitoylated using palmitoylation site prediction tool CSS-PALM4.0 (http://csspalm.biocuckoo.org/). (**E**). Indirective immunofluorescence assays showed that when we introduced a point mutation (C164A) in the DHHC motif of ZDHHC1, NLRP3 expression was no longer increased in HONE1 cells.

To further confirm the stimulation of oxidative stress and ER stress, we applied well-known oxidative stress inhibitor alpha lipoic acids (ALA) together with ER stress inhibitor 4-phenylbutyric acid (4-PBA) on HONE1 cells with or without aberrant ZDHHC1 expression individually 31, 32. Obvious decline of ER stress and pyroptosis markers GRP78, CHOP, NLRP3 and IL-1β was captured on both transcriptional and protein level as the results showed (Figure 6A-B). Thus, we proved the existence of enhanced oxidative and ER insults in ZDHHC1 overexpressing cells, and drop of critical markers indicated that ZDHHC1 indeed exacerbated cellular stress to promote pyroptosis, which contributes to ZDHHC1's anti-oncogenesis. For further clarification, we managed to induce pyroptosis by the means of applying classical pyroptosis trigger reagents, LPS+ATP. It was crystal clear that all indicators in discussion massively upregulated, especially for ZDHHC1 group after stimulations (Figure 6A-B). The introduction of well-established pyroptosis inducer also gave credits to its existence and influence. Outstanding elevated mRNA levels of GSDMD and GSDME told the same story with powerful evidences (Figure 6C). Then, to investigate whether ZDHHC1 promoting NLRP3 expression was dependent of its enzymatic activity, we ectopically expressed wild-type or mutated ZDHHC1 (C164A) in HONE1 cells. The indirect immunofluorescent detection assay demonstrated that NLRP3 can be upregulated by ZDHHC1, but not by ZDHHC1 C164A (Figure 6D-E). Therefore, we concluded that ZDHHC1-induced cytotoxicity at least in part involves pyroptosis and apoptosis through oxidative stress and ER stress signaling pathways.

## Discussion

In this study, we provided evidences showing ZDHHC1 as a potential tumor-suppressor is frequently silenced due to promoter methylation. Promoter methylation of TSGs plays an important role in tumor development 33-37. Mechanism studies suggested that ZDHHC1 inhibits glucose metabolism. Because glucose is a major energy source for cancer cells, interruption of glucose metabolism is regarded as an effective therapeutic strategy 11. ZDHHC1-expressing cells exhibited compromised glucose metabolism, which was associated with decreased expression of rate limiting enzymes and key factors in glucose metabolism. In a previous study, we reported that CYGB reduced expression of GLUT1 and HXK2 and stimulated that of TP53-induced glycolysis and apoptosis regulator (TIGAR) 23. CYGB, a member of the vertebrate globin family 38, has multiple functions including tumor suppression, protection against oxidative stress, and reduction of glucose intake in cancer cells 23, 24, 39-41. The bioinformatics data demonstrated a significant positive correlation between CYGB and ZDHHC1 levels. The recovery experiments demonstrated that CYGB knockdown attenuated ZDHHC1-mediated suppression of proliferation and induction of apoptosis. Thus, it is likely CYGB is one of the key factors downstream of ZDHHC1 in suppressing glucose metabolism and thereby cancer cell growth.

Up to now, there have been 23 distinct zinc finger proteins identified from DHHC-type containing (ZDHHC) family in mammals 20. Different ZDHHC enzymes can act as either oncoproteins or tumor suppressors via regulating specific substrates 42. ZDHHC17 and ZDHHC20 may act as oncoproteins 43, 44, but ZDHHC2 and ZDHHC13 can act as tumor suppressors 45-47. ZDHHC14 can slow tumor growth and induce apoptosis in human embryonic kidney (HEK 293) cells 48. Conversely, ZDHHC14 exhibited oncogenic activity in multiple tumors 49-51. Currently, we first reported that ZDHHC1 acts as a novel tumor suppressor, suppressing tumor cells' proliferation, stimulating apoptosis, and inhibiting cell migration and invasion.

The PPP is important for energy supply in cancer cells. In addition, PPP provides NADPH to cope with excessive ROS for maintenance of redox homeostasis, and ribonucleotide to ensure cell proliferation 4, 52. Our iTRAQ and metabolomic analysis results showed decreased levels of G6PD, a rate limiting enzyme of the PPP 52, and its products upon ZDHHC1 overexpression. Accordingly, it was reported that tumor cells exhibit relatively high G6PD levels 53, which may be due to activation of classical oncogenic pathways such as PI3K/Akt, Ras, and Src 52. Here, we determined that G6PD levels and ribose synthesis were significantly decreased in ZDHHC1 overexpressing cells, suggesting that ZDHHC1 inhibits cancer through the downregulation of G6PD-mediated glucose flux through the PPP. These findings supported a vital role for other metabolic pathways in addition to glycolysis in cancer cell growth. We are also the first to report functional connection between ZDHHC1 and CYGB levels and the PPP.

It is well-known that excessive oxidative stress, which can be caused by a plethora of reactive species, including ROS, reactive nitrogen species (RNS), and reactive sulfur species (RSS), causes damages in DNA, lipids, and proteins that could kill cells 6, 54. Excessive oxidative stress ultimately causes cell death. In the present study, we took multiple measures to estimate oxidative status alteration after ZDHHC1 overexpressing, and observed an increase in the levels of reactive species like H_2_O_2_, ROS and O_2_^-^, whereas no change in total antioxidants upon ZDHHC1 expression. We evidenced urgent oxidative stress in overloaded ZDHHC1 group, which appears beneficial in the cancer inhibition. The unchanged TAC can be interpreted as CYGB offsetting part of the oxidative stress effect.

Our results also showed that ZDHHC1 evoked ER stress, leading to a high proportion of apoptotic and pyroptotic cells. The levels of three well-known ER stress sensors, PKR-related ER kinase (PERK), inositol requiring enzyme-1 (IRE1), and transcription factor 6 (ATF6) 55, were markedly increased, suggesting ZDHHC1 overexpression causes ER stress in a fully-covered fashion. While the main ER stress markers, including X-box-binding protein-1 (XBP1), Jun-N-terminal Kinase (JNK) and C/EBP-homologous protein (CHOP), can stimulate NLRP3 inflammasome to induce pyroptosis 26. NLRP3 inflammasome is not only a crucial link between oxidative/ER stress and pyroptosis, but also pivotal for cleavage and activation of caspase-1 56, and active caspase-1 cleaves gasdermin D (GSDMD) for forming membrane pores that results in pyroptosis 57, 58. Noticing abundant release of IL-1β and IL-18, plus pictures captured under electronical microscope illustrating typical pyrotosis features, in addition to accumulated TNFα and cleaved PARP, ZDHHC1 is therefore a powerful promoter for pyroptosis and apoptosis. Current study managed to exemplify enhanced apoptotic and pyroptotic degree in cells with aberrant ZDHHC 1, appearing to kill cancer cells with joint efforts. Previous studies have revealed ER stress can lead to pyroptosis 26. As a major factor in pyroptosis, GSDME has recently been shown to be able to convert apoptosis into pyroptosis, thereby notably suppressing cancer cell proliferation and improving the beneficial effects of chemotherapy 59. Because GSDME is cleaved by caspase-3 59 and caspase-3 is upregulated in ZDHHC1 overexpressing cells, we postulated caspase-3/GSDME played a role in ZDHHC1-triggered pyroptosis. Furthermore, transcriptional as well as protein levels of pyroptosis biomarkers were remarkedly depleted after application of oxidative and ER stress inhibitors, which confirmed pyroptosis' existence and how oxidative in concert with ER stress pulled the strings to trigger pyroptosis.

In this article, we first argued that pyroptosis is triggered in addition to apoptosis in ZDHHC1 restoration cells. Subsequently, we confirmed the existence of necroptosis. Necroptosis, i.e. programmed necrosis, requiring necrosome and MLKL to complete the procedure. Necrosome is crucial signaling platform comprising the assembly of RIPK1 (receptor-interacting protein [RIP] kinase 1) and RIPK3. Phosphorylated RIPK3 recruits and phosphorylates MLKL, thereafter the activated MLKL (pMLKL) translocates to plasma membrane and conducts perforation 60. In light of membrane pore-formation quality, MLKL is considered the executioner of necroptosis. Investigations have acknowledged onco-suppressive identity of necroptosis in a set of cancer scenarios. And diversified drugs triggering necroptosis in cancer for cure purposes have been discovered and developed as well. Detection of pMLKL is more specific given RIPK1 and RIPK3 are implicated in alternative regulations, and thereby phosphorylation of MLKL is considered key biomarker for necroptosis 30. For proof, we detected pMLKL in paired xenograft nude mice tumor tissues with IHC, and determined a glaring expressing condition within ZDHHC1 group. It should be noted that necroptosis and pyroptosis undergo individual signaling pathways albeit they both fall into programmed necrosis category 28. As results, we manifested elevated p-MLKL levels within ZDHHC1 groups, verifying that necroptosis was significantly induced, concluding that ZDHHC1's onco-suppressive ability is probably described by the induction of both necroptosis and pyroptosis to some degree.

In summary, our experiments confirmed ZDHHC1 is downregulated in several cancer cell lines and multiple tumors by DNA methylation. Furthermore, CYGB is essential for the function of ZDHHC1 as a tumor suppressor in cancer cells. ZDHHC1 led to oxidative and ER stress, thereby stimulating pyroptosis and increasing cancer cell apoptosis. These findings will provide a theoretical basis for the design of new treatments that targets ZDHHC1 and CYGB might have clinical utility as cancer biomarkers, or as the targets of clinical intervention. It should be noted that the ZDHHC1 belongs to the palmitoyl-transferase ZDHHC family, and NLRP3 is a potential substrate for palmitoylation, as predicted by CSS-PALM 4.0 61. It remains to be determined if ZDHHC1 has palmitoyl-transferase activity and whether this activity is involved in its tumor suppression mechanisms.

## Materials and Methods

### Cell lines culture and drug treatment

 Cell lines (HONE1, MCF7, and A549) were provided by ATCC (American Type Culture Collection, Manassas, VA, USA). All cell lines were cultured in RPMI1640 (Gibco-BRL, Karlsruhe, Germany) medium in general use.

LPS and ATP were used for the induction of pyroptosis. LPS and ATP were both purchased from Solarbio Lifesciences (LPS: Cat# L8880; ATP: Cat# IA0590, Tongzhou District, Beijing, China). LPS was added to the culture medium at the concentration of 1 µg/mL for 4 h beforehand, followed by treatment of 5mM ATP for 30 min. Cells were harvested for further use afterwards. 4-PBA and ALA were used as ER stress and oxidative stress inhibitor respectively. Exposed to corresponding reagents at the concentration of 1 mM for 30 min (for 4-PBA), and 20 µM for 2 h (for ALA), pretreated cells were then collected for subsequent use. 4-PBA and ALA were both ordered from Sigma-Aldrich.

### Tumor samples and normal tissues

The tissues involved in this experiment were collected from the First Affiliated Hospital of Chongqing Medical University, including all the primary tumor tissues and adjacent normal tissues. It had been certified by qualified pathologists that the percentage of tumor cells in all tumor samples are over 70. Patient information and consent had been harvested as well. This research was authorized by the Institutional Ethics Committees of the First Affiliated Hospital of Chongqing Medical University (Approval notice #: 2016-61) and complied with the Declaration of Helsinki.

### Quantitative real-time PCR, Semi-quantitative PCR

RNA was isolated from tissues using Trizol according to manufacturer's introductions (Invitrogen, Carlsbad, CA, USA). DNA was extracted with QIAamp DNA mini kit (Qiagen, Hilden, Germany). In RT-PCR, the total volume of each reaction mixture was 10 µL with 2 µL of cDNA, and GAPDH was used as the internal control. The sequence of primers is presented in Table 1. RT-PCR was conducted using the Go-Taq system (Promega, Madison, WI, USA) under the condition of 32 cycles for target genes and 23 cycles for beta-actin. In real-time PCR, all operations complied with the manual (HT7500 System; Applied Biosystems), during which the denaturing/annealing/ extension temperatures were set at 95 °C, 60 °C and 72 °C.

### Bisulfite conversion and methylation-specific PCR (MSP)

Genomic DNA was obtained from cells and tissues. The primers involved were listed in Table 2. AmpliTaq-Gold DNA polymerase was employed to serve the goal of amplifying target genes. After using 2% agar gels for electrophoresis, the results were photographed by a gel imaging system (Bio-RAD Gel Doc XR+, USA). Bisulfite conversion and methylation-specific PCR were conducted as previously described 34, 62.

### Construction of plasmids and stable cell lines

ZDHHC1 and other target genes were inserted into the pEGFP-C2 vector plasmid for ectopic expression. Recombinant plasmids were sequenced prior to transfection with Lipofectamine 2000 (Thermo Fisher Scientific). After transfection, stable cell lines were selected by G418 (300 μg/mL for HONE1, 700 μg/mL for MCF7) and maintained in medium containing half the selected concentration. TRI reagent/protein extraction kit (Thermo Scientific, #23225) was used to perform total RNA/protein extraction. Plasmids contamination was ruled out due to the application of Dnase I (Ambion, Austin, TX, USA). Moreover, we performed RT-PCR to amplify the target genes and used Western blot to confirm the overexpression.

### Cell proliferation assay and colony formation assays

The evaluation of cell growth was performed by cell proliferation assay and colony formation assays. For cell proliferation assay, we seeded cells on 96-well plates and adjusted the cell number at 2000-10000 /well. After allowing cells to attach to the plate, we added CCK-8 (10 μL/well, Beyotime, Shanghai, China) an hour before acquiring the absorbance in 450 nm. The same protocol was used for all time-points.

For colony formation assay, we seeded the cells into 6-well plates at different dilutions. After 10-14 days, 4% paraformaldehyde was used to fix the cells and crystal violet was used to stain the colonies formed by viable cells.

### The soft agar colony formation assay

Cells were seeded on 6-well plates at 37 °C, with the top part of 0.35% agar harboring 1 × 10^3^ cells, along with 1.2% agar in the bottom. All the agars were mixed with RPMI 1640 (additional FBS (fetal bovine serum)). After 3 weeks of incubation, 10 × LEICA microscope was employed for imaging and counting the colonies. Each experiment was repeated three times separately.

### Spheroid-forming assay

The spheroid-forming assay was conducted to examine the effect of ZDHHC1 on tumor cell (MCF7 and HONE1) stemness as described previously 34. Cells stably expressing ZDHHC1 or vector were placed in 6-well plates at a density of 3000 cells per well. The number of tumor spheroids was counted under a microscope (Olympus, Tokyo, Japan) after 14 days of suspension culture.

### Cell cycle analysis

For cell cycle assay by flow cytometry, cells were collected by centrifugation. Next, the supernatant was discarded and the pellet was resuspended in ice-cold 70% ethanol for fixation (30 min at 4 °C). Cells were washed twice with PBS and resuspended in 400 μL PBS and 2.5 μL RNAse (20 mg/mL, DNase free, Sigma-Aldrich). The mixture was incubated at 37 °C for 30 min. 2 μL propidium iodide (final concentration at 5-50 μg/mL) was added before incubating at room temperature for 15-30 min in the dark. Finally, the cells were submitted to flow cytometry analysis.

### AO/EB staining

To assess cellular apoptosis, we applied acridine orange/ethidium bromide (AO/EB) staining. Double-stained cells were subjected to fluorescence microscope imaging (LEICA CTR4000B; Leica Microsystems, Buffalo Grove, IL, USA). Apoptotic rate (%) = (apoptotic cells/total cells) × 100%.

### Annexin V-FITC/PI apoptosis assay

Annexin V-fluorescein isothiocyanate (FITC; BD Biosciences, San Jose, CA) and PI staining were performed according to the manufacturer's protocol. Double-stained cells were analyzed using FACSCalibur^TM^ (BD Biosciences, San Jose, CA). Data were analyzed using the CellQuest^TM^ software (BD Biosciences, San Jose, CA).

### Intracellular ROS

Intracellular ROS was detected by means of DCFH-DA (Beyotime Institute of Biotechnology, Nanjing, China). DCFH-DA was diluted with serum-free medium with 1:1000, and the final concentration was normalized to 10 mol/L. Cells stably expressing or transiently transfected with ZDHHC1 and vector were seeded in 96-well plates (10000 cells per well) with 100 μL of DCFH-DA. Cells were incubated at 37 °C for 30 minutes, and then washed 3 times with serum-free medium. Fluorescence values were measured at 488 nm/525 nm (excitation/emission) with a microplate reader (Multiskan MK3, Thermo Fisher Scientific, Schwerte, Germany).

### Intracellular superoxide levels

Intracellular superoxide levels were detected with dihydroethidium (DHE, Beyotime Institute of Biotechnology, Nanjing, China). Cells stably expressing or transiently transfected with ZDHHC1 and vector were seeded in 96-well plates (10000 cells per well) with 100 μL of DHE. Cells were incubated at 37 °C for 30 minutes, and subsequently washed 3 times with PBS. Fluorescence values were measured at 300 nm/610 nm (excitation/emission) with a microplate reader (Multiskan MK3, Thermo Fisher Scientific, Schwerte, Germany).

### NADP^+^/NADPH ratio

NADPH oxidase activity in the cell was assessed by NADP^+^/NADPH Assay Kit. (Beyotime Institute of Biotechnology, Nanjing, China). Cells stably expressing or transiently transfected with ZDHHC1 and vector were plated at 6-well plates (1×10^6^ cells per well) for 24 h. 200 μL NADP^+^/NADPH extracting solution was added for cell lysis, followed by centrifugation at 12,000 g under 4 °C for 5-10 minutes. 100-200 μL of lysed samples was transferred into a centrifuge tube and heated at 60 °C for 30 minutes to decompose NADP^+^. The samples (100 μL) were transferred into 96-well plates and 100 μL G6PDH was added to each well. The plate was incubated at 37 °C in the dark for 10 minutes to convert NADP^+^ to NADPH. Then 10 μL chromogenic solution was added and the mixture was incubated at 37 °C for 20 minutes. Absorbance values were measured at 450 nm with a microplate reader (Multiskan MK3, Thermo Fisher Scientific, Schwerte, Germany).

### Total antioxidant capacity

Total antioxidant capacity in the cell was assessed by T-AOC Assay Kit (Beyotime, institute of biological engineering, Nanjing, China).Cells stably expressing or transiently transfected with ZDHHC1 and vector were plated at 6-well plates (1×10^6^ cells per well) for 24 h. Cells were collected with cell scraper, and added with 200 μL PBS. Ultrasound was used to fragment cells to release antioxidant compounds, followed by a centrifugation at 12,000g under 4 °C for 5-10 minutes to harvest supernatant. The samples were placed in 96-well plates with 100 μL each well. ABTS working liquid and peroxidase were added. After 6 minutes reaction at room temperature, absorbance values were measured at 405 nm with the microplate reader (Multiskan MK3, Thermo Fisher Scientific, Schwerte, Germany).

### iTRAQ proteomics and GC-MS metabolomics analyses

iTRAQ proteomics and GC-MS metabolomics analyses were conducted as described previously 23.

### Wound healing and Transwell assays for cell migration and invasion

The motility of cells was measured by wound healing and Transwell assays as previously described 34. Briefly, 5 × 10^5^ cells were seeded in 6-well plates and cultured overnight. On the next day, confluent cells were scratched with sterilized tips, and PBS was used to wash the detached cells. Serum-free culture medium was added. Photos were documented every 12 h until cells were confluent again. For the Transwell migration assay, cells were seeded in the Transwell chamber with serum-free medium and the chambers were placed in 24-well plates with regular medium. For Transwell invasion experiment, Matrigel was placed on the upper surface of chamber ahead of seeding. After the appropriate amount of time, cells on the lower side of the membrane were fixed with methyl alcohol and then stained with Giemsa solution. Nine fields were captured for each chamber under a microscope at ×100 magnification.

### Indirect immunofluorescence staining

Cells were seeded onto glass coverslips, which were further fixed with 4% paraformaldehyde. Secondly, we utilized 0.1% Triton X-100 for permeabilizing the cell membrane. To prevent non-specific antibody conjugation, the cells were incubated with 1% bovine serum album in PBS at room temperature for 1h. Subsequently, the primary antibodies were added and the coverslips were incubated at 4 °C overnight prior to the incubation with secondary antibodies at room temperature for 1 h. Next, DAPI (4′,6-diamidino-2-phenylindole) was used to for nucleic counterstaining, which lasted 5 min at room temperature. Images were obtained under a fluorescence microscope.

### Immunohistochemistry (IHC)

Immunohistochemistry was conducted obeyed by previous published protocol 33. The following antibodies were selected for detection: Ki67 (ab16667, Abcam), PCNA (sc-56,Santa Cruz biotechnology); pMLKL (ab196436, Abcam).

### Tumor xenograft model in nude mouse

The breeding process was completely compliant with the guidelines of the Experimental Animal Center of Chongqing Medical University. Cells (5×10^6^) were re-suspended in 0.1 mL PBS and injected subcutaneously at the lower back of each mouse (n = 6). During the following 30 days, a vernier caliper was utilized to measure the length and width of the tumor every three days. Tumor volume was calculated using the formula: volume = length × width^2^ × 0.52.

### Terminal deoxynucleotidyl transferase (TUNEL) analyses

 TUNEL analysis is a biotechnology of examining apoptotic cells in tissues. We used this method to determine the apoptosis status of xenograft tumor tissues. TUNEL detection kit was from Beyotime (Beyotime Institute of Biotechnology, Nanjing, China), and the results were observed and photographed under a fluorescence microscope.

### Western blot

Western blotting was conducted as previously described 34. The following primary antibodies were used in this study: GRP78 (# 3177; Cell Signaling Technology), GRP94 (#3196-1; Epitomics), pERK1/2 (#sc-377400, Santa Cruz Biotechnology), p65 (#sc-8008; Santa Cruz Biotechnology), CHOP(#2895; Cell Signaling Technology), cleaved PARP (#sc-56196, Santa Cruz Biotechnology), NLRP3 (#sc-134306; Santa Cruz Biotechnology), HXK2 (#sc-374091, Santa Cruz Biotechnology), IL-1β (#sc-12742; Santa Cruz Biotechnology), IL-18 (#sc133127; Santa Cruz Biotechnology), p-eIF2a (#33981; Cell Signaling Technology), Casp1 (#YH050707C; Epitonics), ZDHHC1 (#AP10315a; Abgent), E-cadherin (#sc8426; Santa Cruz Biotechnology), Occludin (#TA306787; Origene), N-cadherin (#610920; BD transduction laboratories), Vimentin (#5741s; Cell Signaling Technology), G6PD (#12263s; Cell Signaling Technology), Casp3 (#9664; Cell Signaling Technology), Casp7 (#8438; Cell Signaling Technology), GLUT1(#sc-377228; Santa Cruz Biotechnology), beta-actin (#sc8432; Santa Cruz Biotechnology), GAPDH (#sc-47727; Santa Cruz Biotechnology).

### Transmission electron microscopy

Cells were fixed in 2.5% glutaraldehyde at 4 °C overnight and 1% osmium tetroxide at room temperature successively, followed by dehydration in ethanol. Following incubation in acetone for 20 min, the cells were treated with 50% (1 h), 75% (3 h) and 100% (overnight) epoxy resin and heated at 70 °C overnight. The embedded cells were sliced to ultrathin sections (70 nm) using an MT-5000 Sorvall microtome (Sorvall; Thermo Fisher Scientific, Inc.). Sections were stained with 3% uranyl acetate and 3% lead citrate for 15 min at room temperature and were visualized with a transmission electron microscope system.

### Statistical analyses

All data were processed using the SPSS software VERSION.6 (Chicago, IL, USA). Student's T test, the chi-square test, together with Fisher's exact test were used according to the specific. Statistical significance was acknowledged when *p* <0.05.

## Supplementary Material

Supplementary figures.

## Figures and Tables

**Table 1:** List of expression primers used in this study

PCR	Primer	Sequence (5'-3')	Product size (bp)	PCR Cycles	Annealing temperature (°C)
RT-PCR	ZDHHC1F	CCTGAGAAGAGTGTGTGGAC	277bp	35	55
	ZDHHC1R	CATCTGCTGGATCGATGGAG			
	β-actinF	TCCTGTGGCATCCACGAAACT	315bp	23	55
	β-actinR	GAAGCATTTGCGGTGGACGAT			
qRT-PCR	NLRP3F	ACAGCCACCTCACTTCCAG	168bp		60
	NLRP3R	CCAACCACAATCTCCGAATG			
	NFKBF	GGGGCACGATTGTCAAAGA	119bp		60
	NFKBR	GGGGACTACGACCTGAATGC			
	IL1BF	CTCCAGGGACAGGATATGGA	194bp		60
	IL1BR	TTCTGCTTGAGAG GTGCTGA			
	IL18F	ACAGCTTCGGGAAGAGGAAAGGAA	127bp		60
	IL18R	TGTCTTCTACTGGTTCAGCAGCCA			
	CHOPF	AGAGATGGCAGCTGAGTCAT	177bp		60
	CHOPR	GCAGGGTCAAGAGTGGTGAA			
	GRP78F	AACACGGTCTTTGACGCCAAG	143bp		60
	GRP78R	GTTTGCCCACCTCCAATATCAAC			
	GRP94F	AGAACCTGCTGCATGTCACAG	181bp		60
	GRP94R	GCGGAATAGAAACCGACACCA			
	EIF2AF	TACTGCACTCCTTCGACCTC	114bp		60
	EIF2AR	GTATCCCAGCTGTGCCATCT			
	ATF4F	CGGGACAGATTGGATGTTGGAG	182bp		60
	ATF4R	TTGGGTTCACCGTCTGGGG			
	PERKF	TATGGACTCAGTGCATATAGTGG	162bp		60
	PERKR	CTTCTCATTGCCACTGCGAG			
	ATF6F	CACAGACACTGATGAGCTGCA	129bp		60
	ATF6R	CAGATTTGGTTGTTGATGTCCCA			
	XBP1F	CCTGGTTGCTGAAGAGGAGG	136bp		60
	XBP1R	CCATGGGGAGATGTTCTGGAG			
	IRE1F	GCAGGACATCTGGTATGTTAT	137bp		60
	IRE1R	GCTACATGGTGATGGTGTATTC			
	TNFaF	GCAGTCAGATCATCTTCTCG	140bp		60
	TNFaR	TTATCTCTCAGCTCCACGCC			
	Caspase1F	TACAGACAAGGGTGCTGAAC	234bp		60
	Caspase1R	GTACTCCTTGAGAGTCTTGC			
	β-actinF	GTCTTCCCCTCCATCGTG	113bp		60
	β-actinR	AGGGTGAGGATGCCTCTCTT			

Note. RT-PCR: Semiquantitative real-time PCR, qPCR: quantitative real-time PCR.

**Table 2:** List of MSP primers used in this study

MSP	Primer	Sequence (5'-3')	Product size (bp)	PCR Cycles	Annealing temperature (°C)
MSP	ZDHHC1m1	ATTTATCGGTTTAGTTTTCGTTC	112bp	40	60
	ZDHHC1m2	CTCCCCGAAACAACCGCG			
	ZDHHC1u1	GATTTATTGGTTTAGTTTTTGTTT	114bp	40	58
	ZDHHC1u2	CCTCCCCAAAACAACCACA			
BGS	ZDHHC1BGSF	TTAAGAGGGTTTTTGGTAAGTT	396bp	40	60
	ZDHHC1BGSR	TAAACCCTACCAAAATCAACC			

Note. MSP: methylation-specific PCR, BGS: Bisulfite genomic sequencing.

## Abbreviations

PPP: pentose phosphate pathway
GC-MS: gas chromatography-mass spectrometry
iTRAQ: isobaric tags for relative and absolute quantitation
ER: endoplasmic reticulum
ROS: reactive oxygen species
AO/EB: applied acridine orange/ethidium bromide
DHE: dihydroethidium
DCFH-DA: oxidation-sensitive fluorescent probe
TAC: Total antioxidant capacity
EMT: epithelial-mesenchymal transition
PCA: Principal component analysis
PLS-DA: partial least squares discriminant analysis
OPLS-DA: orthogonal partial least squares discriminant analysis
FBS: fetal bovine serum
IHC: immunohistochemistry
PBS: phosphate buffered saline
qPCR: quantitative real-time PCR
RT-PCR: Semiquantitative PCR
A+T: 5-Aza-2′-deoxycytidine and trichostatin A (TSA)
MSP: methylation-specific polymerase chain reaction
BGS: Bisulfite genomic sequencing
DAPI: 4'-6-diamidino-2-phenylindole
PI: propidium iodide
M: methylated
U: unmethylated
