# Supplementary material for: DNA methylation downregulated ZDHHC1 suppresses tumor growth by altering cellular metabolism and inducing oxidative/ER stress-mediated apoptosis and pyroptosis
Source: Theranostics. 2020 Jul 25;10(21):9495–511. doi: 10.7150/thno.45631 (PMC7449911; doi:10.7150/thno.45631)
Supplement: Supplementary file 1 — Supplementary figures. [file thnov10p9495s1.pdf]

Supplementary figure legends

Figure S1.

***ZDHHC1* inhibited growth, migration and invasion of MCF7 and HONE1 cells.**

(A). Representative images of colony-formation assays. (B). Flow cytometry analysis of cell cycle progression. (C). Flow cytometry analysis of cellular apoptosis. Vector and *ZDHHC1* transfected MCF7 and HONE1 cells were double stained with Annexin-V-FITC and PI for apoptosis measurement. (D). Morphology of MCF7 cells undergoing apoptosis were observed under electronic microscopy. (E). Representative images of wound-healing assays conducted with MCF7 and HONE1 overexpressing *ZDHHC1* or Vector. Photos were taken at 0h and 48h after the wound was made. (F). Representative images of Transwell assays measuring cell migration ability, using all two cells transfected with Vector or *ZDHHC1* 24h after seeding. (G). Representative images of Transwell assays measuring cell invasion ability, using MCF7 and HONE1 cells transfected with Vector or *ZDHHC1*.

Figure S2.

Knocked down *ZDHHC1* enhanced growth, migration and invasion of A549 cell.

(A-B). Knocked down *ZDHHC1* expression enhanced A549 cell growth. A549 cell transfected with sh*ZDHHC1* or empty vector were used for colony formation assay and CCK8 assay. (C). Flow cytometry analysis of cellular apoptosis. Vector and *ZDHHC1* knocked down in A549 cell were double stained with Annexin-V-FITC and PI for apoptosis measurement. (D). Flow cytometry analysis of cell cycle progression. (E).Transwell assays measuring cell migration and invasion ability, using A549 cell transfected with Vector or sh*ZDHHC1* 24h after seeding.

Figure S3.

Expression proteomic and metabolomic analyses of *ZDHHC1* overexpression in MCF7 cells.

(A). Enrichment of differential-expressed proteins categorized by function.(B). KEGG Pathway analysis showed the expression of proteins involved in multiple pathways, among which metabolic pathway are greatest altered by ZDHHC1. (C). Effects of ectopic expression of CYGB and ZDHHC1 had on multiple proteins. (D). The Venn diagram showed an overlap of 18 proteins were regulated by both ZDHHC1 and CYGB.

Figure S4.

Supplementary Figure 4. PPI network of protein interaction was constructed from bioinformatics.

Figure S5.

***ZDHHC1* promote *CYGB* expression and inhibit carbohydrate metabolites in MCF7 cells.**

(A).The positive correlation between *ZDHHC1* and *CYGB* was confirmed by bioinformatics (B). Verification of CYGB expression was increased in ZDHHC1 cells by qPCR .(C).Pathway analysis of differential metabolites confirmed by gas chromatographymass spectrometry metabolomics assay. Data was analyzed by MetaboAnalyst software. (D).Intracellular levels of glucose and its metabolites confirmed by GC-MS metabolomics analyses. (E). Expression of *ZDHHC1* decreased GLUT1, HXK2, and G6PD identified by Western blot in vector- and *ZDHHC1*- transfected MCF-7 and HONE1 cells.

Figure S1

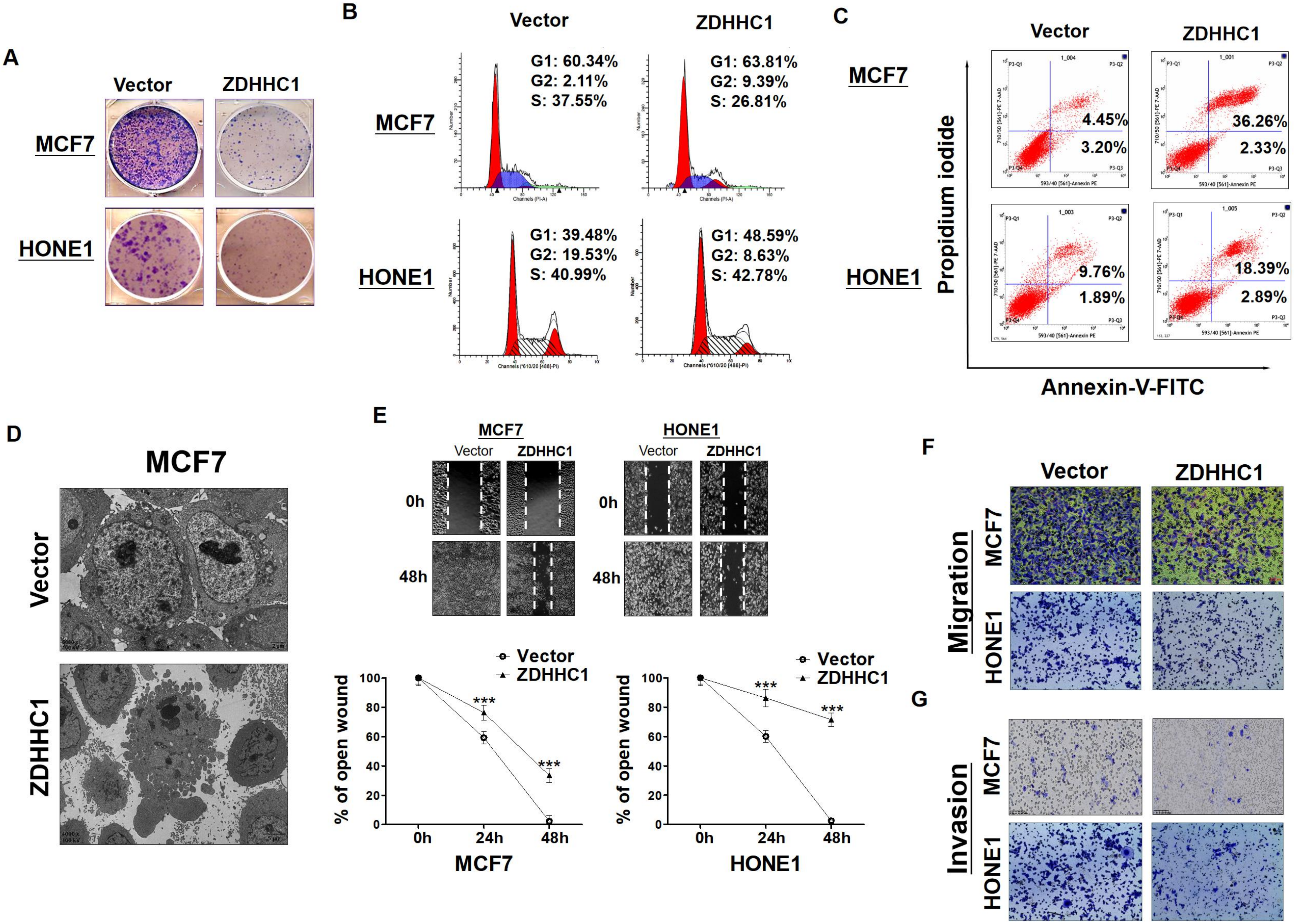

A
Vector
ZDHHC1
MCF7
HONE1
B
Vector
ZDHHC1
MCF7
HONE1
G1: 60.34%
G2: 2.11%
S: 37.55%
G1: 63.81%
G2: 9.39%
S: 26.81%
G1: 39.48%
G2: 19.53%
S: 40.99%
G1: 48.59%
G2: 8.63%
S: 42.78%
C
Vector
ZDHHC1
MCF7
HONE1
Propidium iodide
Annexin-V-FITC
4.45%
3.20%
36.26%
2.33%
9.76%
1.89%
18.39%
2.89%
D
MCF7
Vector
ZDHHC1
E
MCF7
HONE1
Vector
ZDHHC1
0h
48h
Vector
ZDHHC1
% of open wound
0h
24h
48h
***
MCF7
HONE1
F
Vector
ZDHHC1
Migration
MCF7
HONE1
G
Invasion
MCF7
HONE1

Figure S2

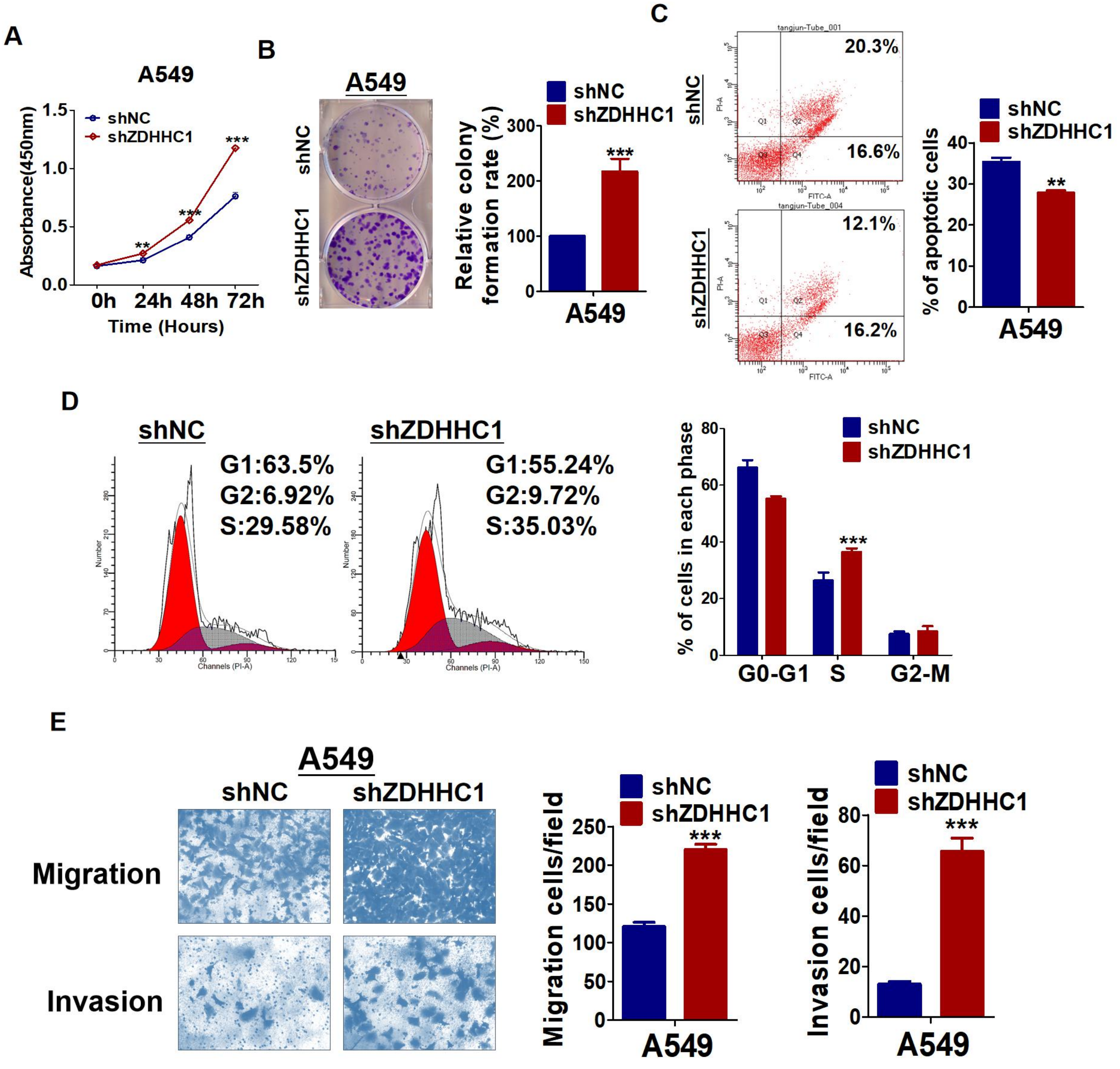
A
A549
Absorbance(450nm)
shNC
shZDHHC1
Time (Hours)
0h 24h 48h 72h
B
A549
Relative colony formation rate (%)
C
20.3%
16.6%
12.1%
16.2%
% of apoptotic cells
D
G1:63.5%
G2:6.92%
S:29.58%
G1:55.24%
G2:9.72%
S:35.03%
Channels (PI-A)
% of cells in each phase
G0-G1
S
G2-M
E
Migration
Invasion
Migration cells/field
Invasion cells/field

Figure S3

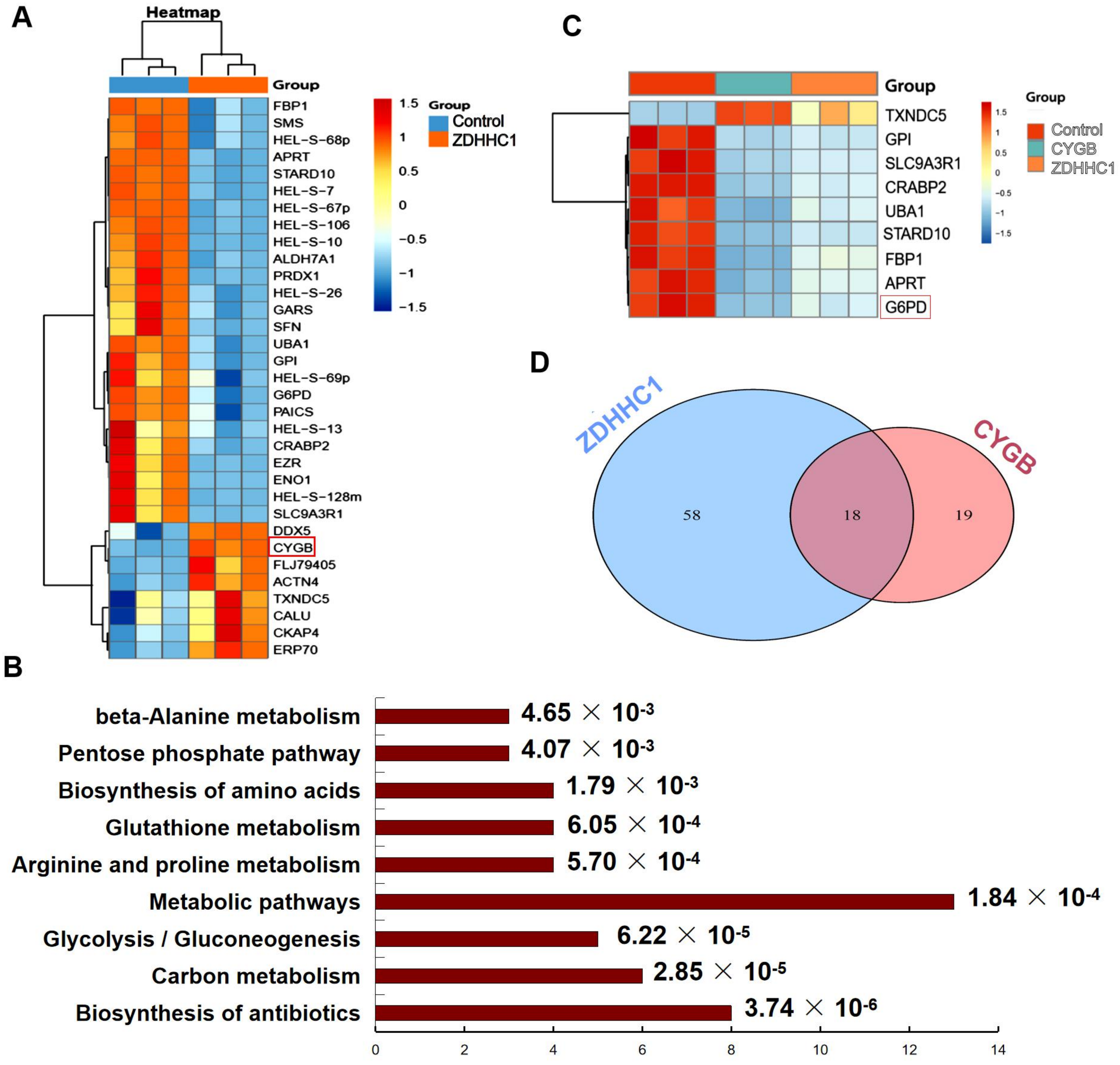
A
Heatmap
Group
FBP1
SMS
HEL-S-68p
APRT
STARD10
HEL-S-7
HEL-S-67p
HEL-S-106
HEL-S-10
ALDH7A1
PRDX1
HEL-S-26
GARS
SFN
UBA1
GPI
HEL-S-69p
G6PD
PAICS
HEL-S-13
CRABP2
EZR
ENO1
HEL-S-128m
SLC9A3R1
DDX5
CYGB
FLJ79405
ACTN4
TXNDC5
CALU
CKAP4
ERP70
1.5
1
0.5
0
−0.5
−1
−1.5
Group
Control
ZDHHC1
B
beta-Alanine metabolism
Pentose phosphate pathway
Biosynthesis of amino acids
Glutathione metabolism
Arginine and proline metabolism
Metabolic pathways
Glycolysis / Gluconeogenesis
Carbon metabolism
Biosynthesis of antibiotics
4.65 × 10-3
4.07 × 10-3
1.79 × 10-3
6.05 × 10-4
5.70 × 10-4
1.84 × 10-4
6.22 × 10-5
2.85 × 10-5
3.74 × 10-6
0
2
4
6
8
10
12
14
C
Group
TXNDC5
GPI
SLC9A3R1
CRABP2
UBA1
STARD10
FBP1
APRT
G6PD
1.5
1
0.5
0
−0.5
−1
−1.5
Group
Control
CYGB
ZDHHC1
D
ZDHHC1
CYGB
58
18
19

Figure S4

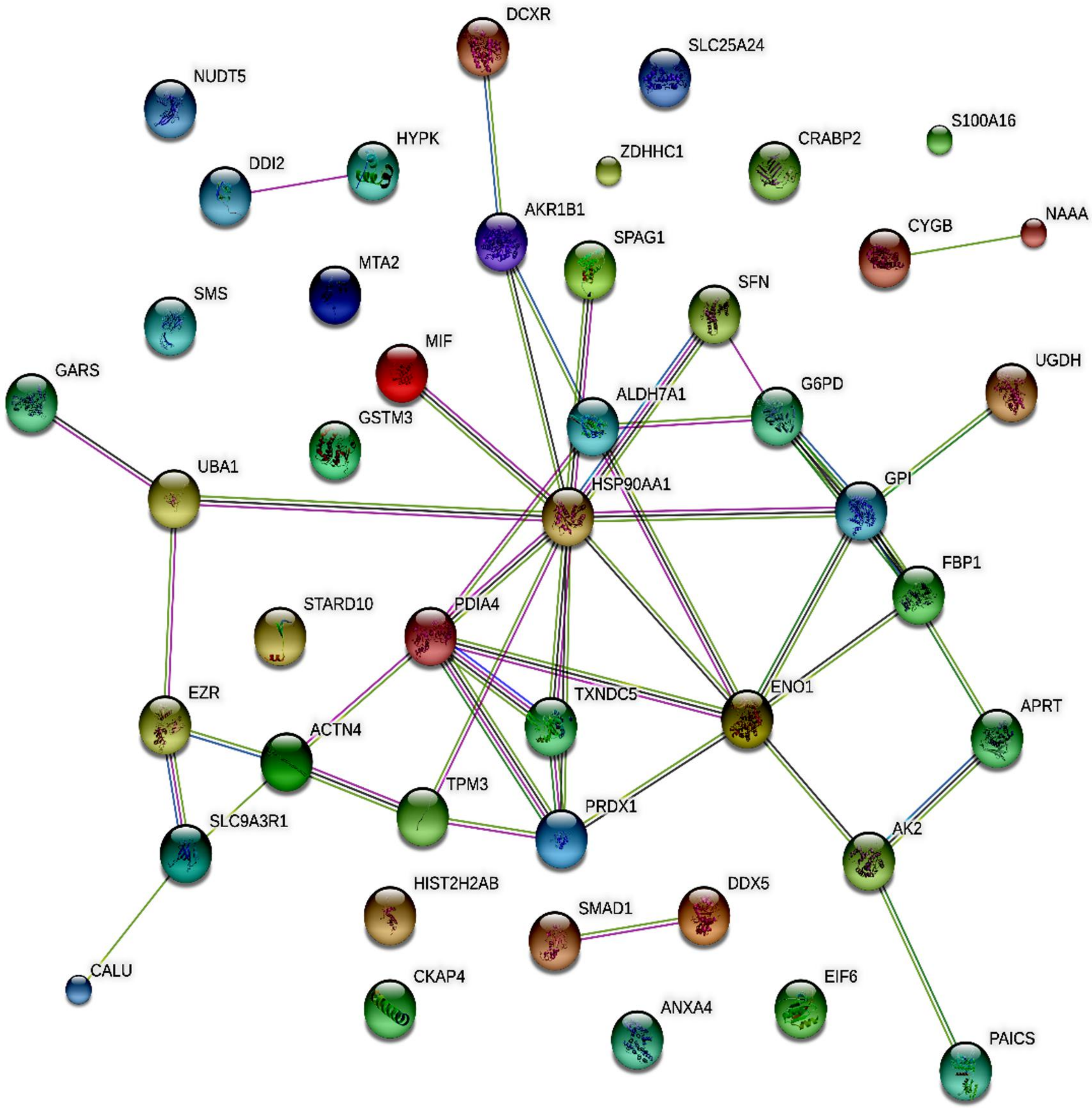
DCXR
SLC25A24
NUDT5
HYPK
DDI2
ZDHHC1
CRABP2
S100A16
AKR1B1
CYGB
NAAA
SPAG1
MTA2
SMS
SFN
MIF
UGDH
GARS
ALDH7A1
G6PD
GSTM3
UBA1
HSP90AA1
GPI
FBP1
STARD10
PDIA4
ENO1
EZR
TXNDC5
APRT
ACTN4
TPM3
PRDX1
SLC9A3R1
AK2
HIST2H2AB
DDX5
SMAD1
CALU
CKAP4
EIF6
ANXA4
PAICS

Figure S5

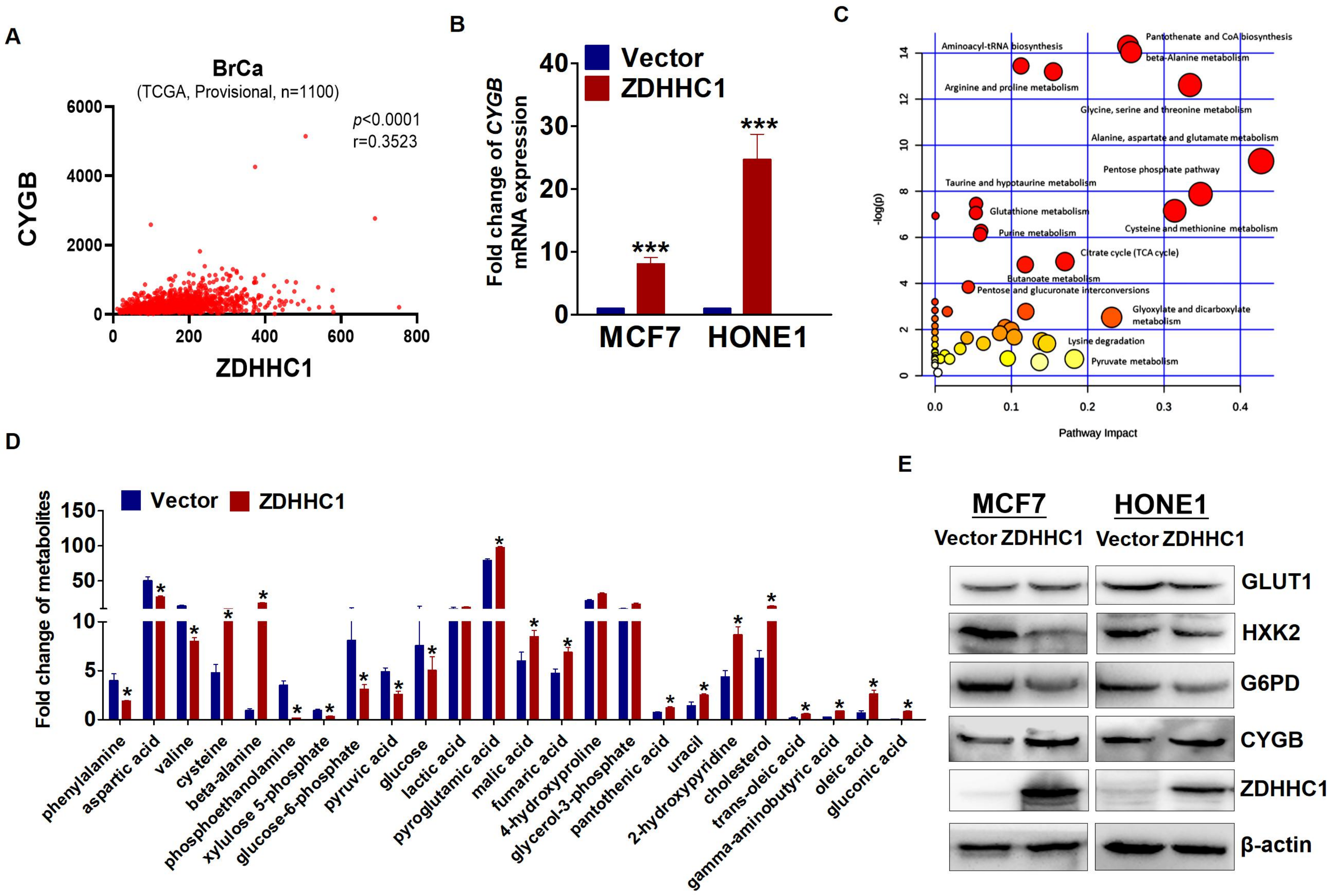
A
BrCa
(TCGA, Provisional, n=1100)
p<0.0001
r=0.3523
CYGB
ZDHHC1
6000
4000
2000
0
0
200
400
600
800
B
Vector
ZDHHC1
Fold change of CYGB mRNA expression
40
30
20
10
0
***
***
MCF7
HONE1
C
Pantothenate and CoA biosynthesis
Aminoacyl-tRNA biosynthesis
beta-Alanine metabolism
Arginine and proline metabolism
Glycine, serine and threonine metabolism
Alanine, aspartate and glutamate metabolism
Pentose phosphate pathway
Taurine and hypotaurine metabolism
Glutathione metabolism
Purine metabolism
Cysteine and methionine metabolism
Citrate cycle (TCA cycle)
Butanoate metabolism
Pentose and glucuronate interconversions
Glyoxylate and dicarboxylate metabolism
Lysine degradation
Pyruvate metabolism
-log(p)
Pathway Impact
0.0
0.1
0.2
0.3
0.4
D
Vector
ZDHHC1
Fold change of metabolites
150
100
50
10
5
0
phenylalanine
aspartic acid
valine
cysteine
beta-alanine
phosphoethanolamine
xylulose 5-phosphate
glucose-6-phosphate
pyruvic acid
glucose
lactic acid
pyroglutamic acid
malic acid
fumaric acid
4-hydroxyproline
glycerol-3-phosphate
pantothenic acid
uracil
2-hydroxypyridine
cholesterol
trans-oleic acid
gamma-aminobutyric acid
oleic acid
gluconic acid
E
MCF7
HONE1
Vector ZDHHC1
Vector ZDHHC1
GLUT1
HXK2
G6PD
CYGB
ZDHHC1
β-actin
